# Successful Utilization of High-Flux Hemodialysis for Treatment of Vancomycin Toxicity in a Child

**DOI:** 10.1155/2011/678724

**Published:** 2012-01-29

**Authors:** Timothy Stidham, Pamela D. Reiter, Douglas M. Ford, Gary M. Lum, Joseph Albietz

**Affiliations:** ^1^Pediatric Intensive Care Unit, Section of Critical Care Medicine, Children's Hospital Colorado and University of Colorado at Denver, 13123 East 17th Avenue, Mail Stop 8414, Aurora, CO 80045, USA; ^2^Pediatric Intensive Care Unit, Department of Pharmacy, Center for Pediatric Medicine, Children's Hospital Colorado and University of Colorado Denver School of Pharmacy, 13123 East 17th Avenue, Campus Box 375, Aurora, CO 80045, USA; ^3^Department of Nephrology, University of Colorado at Denver and Children's Hospital Colorado, 13123 East 16th Avenue, Campus Box B328, Aurora, CO 80045, USA; ^4^Pediatric Intensive Care Unit, Section of Critical Care Medicine, University of Colorado at Denver and Children's Hospital Colorado, 13123 East 17th Avenue, Campus Box B530, Aurora, CO 80045, USA

## Abstract

Vancomycin is routinely used for empiric antibiotic therapy in children. Higher-serum-concentration targets for serious infections are now being recommended. This recommendation may result in aggressive dosing with increased potential for toxicity. We report a case of a pediatric patient who developed vancomycin toxicity and associated oliguric renal failure who was treated effectively with high-flux hemodialysis for vancomycin toxicity, clearing serum concentrations of vancomycin by over 75% in only 6 hours (213.2 mcg/mL to 51.8 mcg/mL) with subsequent return to baseline renal function and without adverse sequelae. While not historically considered a viable option for drug removal in cases of toxicity, new high-flux hemodialysis techniques can remove significant percentages of vancomycin in short periods of time.

## 1. Background

Vancomycin is a narrow-spectrum tricyclic glycopeptide antibiotic with bactericidal activity against a variety of gram-positive organisms including *Staphylococcus epidermidis, Staphylococcus aureus* (both methicillin-sensitive (MSSA) and- resistant strains (MRSA)), streptococci, enterococci, and diphtheroids. It is often selected as part of empiric therapy for presumed serious bacterial infections in children. Since bacterial killing by vancomycin is considered time dependent, it is important to maintain vancomycin serum concentrations above the minimum inhibitory concentration (MIC) of the organism for at least 40–50% of a dosing interval [1]. Historically, MIC values for susceptible organisms were less than 2 mcg/mL, making a target serum trough concentration of 5–10 mcg/mL acceptable for therapeutic success. Today, however, the MIC values are higher, in part from the increase in resistant microorganisms. Vancomycin dosing recommendations, in turn, have been increased in order to achieve higher serum concentrations. Recent adult guidelines for pneumonia (hospital acquired and ventilator associated) and meningitis now recommend achieving a vancomycin serum trough concentration of 15–20 mcg/mL [2–4]. Although pediatric recommendations are not included in these guidelines, higher concentrations of vancomycin are now being targeted in children on a routine basis. These higher serum concentrations may increase the likelihood of vancomycin-associated nephrotoxicity. Historically, dialysis has not been a highly effective therapy in the treatment of vancomycin toxicity. We report a case of vancomycin- associated nephrotoxicity, successfully treated with high-efficiency dialysis, in a child requiring high-dose therapy to achieve serum trough concentrations within the newly recommended target of 15–20 mcg/mL.

## 2. Case Presentation

A seven-year-old female with congenital hydrocephalus requiring placement of a ventriculoperitoneal shunt (VPS) at 18 months of age with recent shunt revision was admitted to our tertiary care children's hospital with cellulitis around the abdominal incision, malaise, poor enteral intake, and headache. An operative procedure was performed to externalize her shunt. Wound cultures from the incision grew MRSA and *Streptococcal anginosus* (milleri), and cultures from her shunt tip grew *Enterobacter cloacae*. She was initially placed on ceftriaxone 50 mg/kg intravenous (IV) every 12 hours and vancomycin 15 mg/kg IV every 8 hours with a goal trough vancomycin concentration of 15 mcg/mL and goal peak of 35–40 mcg/mL. Blood was collected and revealed a trough of 3.7 mcg/mL and a peak of 16.9 mcg/mL—both below target. The vancomycin regimen was increased to 25 mg/kg IV every 6 hours with a resulting trough of 11.2 mcg/mL and peak of 25.2 mcg/mL (end of infusion peak = 34 mcg/mL). While the peak concentration was adequate for this central nervous system infection, the trough concentration was below target. Vancomycin was further increased to 29 mg/kg IV every 6 hours. Subsequent serum concentrations with the third dose revealed a therapeutic trough of 15 mcg/mL and a peak of 34.6 mcg/mL (end of infusion peak = 50 mcg/mL). Due to the requirement of this aggressive dosing regimen, surveillance serum creatinine was requested every 2-3 days. Four days later, the child's urine output had fallen from 2.4 mL/kg/hr to below 1 mL/kg/hour, and she showed clinical signs of fluid overload with peri-orbital edema and ascites. Laboratory analysis revealed a serum creatinine of 3.47 mg/dL (baseline = 0.28 mg/dL on admission). Serum potassium was within normal limits (3.8 mmol/L). Serum bicarbonate was low (15 mmol/L). Vancomycin was held and a 9-hour postdose concentration was 213 mcg/mL. Upon review of her medication profile, she had been exposed to multiple doses of ketorolac and ibuprofen for postoperative pain management.

**Figure 1:**
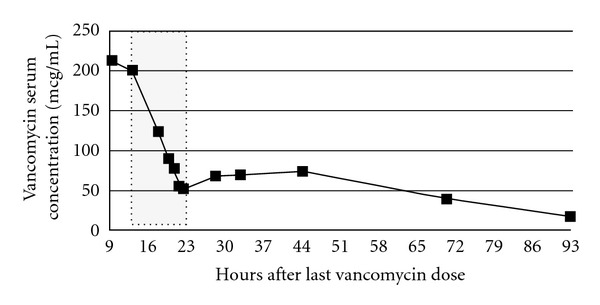
Concentration-time curve of vancomycin in a 7-year-old child. Initial concentration was collected nine hours after a dose. Shaded area indicates time of active high-flux hemodialysis therapy. Total dialysis time = 6 hours.

The patient's acute oliguric renal insufficiency and excessive vancomycin concentration prompted a renal consult. A renal ultrasound was obtained that showed bilateral increased renal echogenicity consistent with medical renal disease and demonstrated no anatomic renal anomaly to explain her acute renal insufficiency. Her vital signs upon admission to the pediatric intensive unit were notable only for slight tachycardia with heart rate 132, blood pressure 106/70, respiratory rate 20, and 100% saturated on room air. Access for hemodialysis was obtained using a Dual Lumen 9 French, 12 cm central venous catheter placed into the right femoral vein. Hemodialysis was performed using a F40S hi-flux polysulfone dialyzer, 140 Na, 4 K and 35 HCO_3_ bath, and a blood flow rate of up to 180 mL/min was achieved. During dialysis she also received furosemide to promote urine output and decrease intrarenal vancomycin accumulation. Serial vancomycin concentrations were collected during dialysis (Figure 1), and dialysis was stopped after 6 hours when a concentration of less than 50 mcg/mL was achieved. The patient tolerated dialysis well with periodic albumin and saline support. Her postdialysis vital signs were stable (blood pressure = 91/40 mmHg; heart rate = 141 beats per minute), 426 mL of fluid was removed, and total of 150 mL fluids (albumin and saline) infused for net positive fluid balance of 276 mL. Serum creatinine values also declined during dialysis from a peak of 3.59 mg/dL to a nadir of 1.09 mg/dL. 

 After completion of the dialysis procedure, she was transferred back to the medical floor in stable condition. Serum vancomycin and creatinine concentrations were followed every 12 hours for 2 days and then daily until within normal limits for age. There was a noticeable redistribution effect of vancomycin following cessation of dialysis (Figure 1) with a resulting rise in vancomycin concentration to a second peak of 73.9 mcg/mL 22 hours after stopping dialysis. Serum vancomycin levels steadily declined over the next 3 days. Likewise, serum creatinine values initially increased after completion of dialysis but then declined over the following days to 0.49 mg/dL prior to discharge. Antibiotic therapy was changed to linezolid and levofloxacin. 13 days after admission to the PICU, her external ventricular drain was removed and replaced with a VPS. Because of the potential for ototoxicity with high vancomycin concentrations, a hearing evaluation was performed. The auditory brainstem responses demonstrated normal bilateral peripheral hearing sensitivity. She was ultimately discharged home once recuperated from her surgery and required no additional renal support.

## 3. Discussion

Vancomycin is eliminated by the kidneys via glomerular filtration [5]. When used alone, vancomycin is considered minimally nephrotoxic—especially when targeting serum trough concentrations of 5–10 mcg/mL. The risk of nephrotoxicity, however, increases with elevated serum trough concentrations, prolonged duration of drug exposure, concomitant use of other nephrotoxic medications (e.g., aminoglycosides, radiocontrast dye, furosemide, amphotericin B and nonsteroidal anti-inflammatory agents), and extracellular fluid contraction [6, 7]. A recent evaluation of vancomycin-associated nephrotoxicity identified nine studies that reported elevated serum trough concentrations (≥15 mcg/mL) as a significant predictor of nephrotoxicity [8]. The influence of vancomycin dose and serum concentration on the risk of developing nephrotoxicity in children was studied among 167 children (age: 1 week–19 years) [9]. These investigators reported an overall nephrotoxicity rate of 14%—which was significantly higher in children who attained a serum trough concentration above 15 mcg/mL, compared to those who achieved a trough concentration below 15 mcg/mL (28% versus 7.3%, *P* = 0.0001). It is important to note that most reports describing vancomycin-associated nephrotoxicity have been unable to firmly establish causation due to the inability to document the temporal relationship of the onset of renal insufficiency and vancomycin serum levels due to the inability to completely rule out that renal function declined before the rise in serum vancomycin concentration. In our case, it seems more likely that vancomycin nephrotoxicity preceded renal dysfunction because our patient had ample evidence of highly efficient vancomycin clearance early in her course and, other than medication administration, sustained no other discernable renal insult. Though the initiating factor is uncertain, once acute renal insufficiency occurs, a positive feedback loop is established, further compromising renal function. In our case, the nephrotoxicity was likely compounded by exposure to multiple doses of nonsteroidal anti-inflammatory agents contemporaneous to vancomycin. Though most cases of vancomycin-associated nephrotoxicity resolve upon discontinuation of the medication, the markedly high serum vancomycin levels, compounded by impaired clearance due to oliguria, further increased the risk of persistent permanent renal injury and ototoxicity and warranted augmented clearance through high-flux hemodialysis.

Prior to the introduction of high-flux hemodialysis membranes for renal replacement therapies, a variety of less efficient modalities for the treatment of vancomycin toxicity have been attempted. In patients with markedly high plasma vancomycin concentrations but preserved renal function, vancomycin elimination can be enhanced by aggressive diuresis alone [10]. When more aggressive drug removal has been indicated, especially in the presence of renal dysfunction, conventional membrane dialysis and charcoal hemoperfusion have been attempted but with limited success. Standard membrane dialysis is largely ineffective and much slower in clearing higher mass molecules such as vancomycin (molecular weight approximately 1448 Daltons). Alternative methods to remove vancomycin from the intravascular space have included charcoal hemoperfusion (CH), gastric dialysis using multidose activated charcoal and exchange transfusion, with CH apparently the more efficient of the two [11]. While CH can remove approximately 40% of vancomycin from the intravascular compartment, this technique is accompanied by many adverse effects including hypocalcemia, hypothermia, hemodynamic compromise, and thrombocytopenia [12]. Attempts to ameliorate these adverse effects of CH with the use of simultaneous HD have had minimal success. Additionally, the shelf life of the CH membrane is quite short, resulting in increased cost. As a result, CH has largely been abandoned as a viable therapy for vancomycin toxicity.

High-flux hemodialysis is defined by increased clearance of middle-weight molecules (defined by beta-2 microglobulin clearance over 20 mL/min). As compared to standard hemodialysis, the membranes typically have a higher ultrafiltration coefficient and are composed of either synthetic or cellulose membranes. Disadvantages to the use of high-flux hemodialysis include the need for an automated ultrafiltration control system with large extracorporeal circuit, higher cost of the machines compared to conventional dialysis machines, and like other forms of hemodialysis reliable central venous access is a necessity [13]. Despite these drawbacks, the larger pore size of high-flux membranes allows for enhanced elimination of larger molecules and protein-bound molecules, previously not amenable to hemodialysis.

Our report highlights the safety and effectiveness of dialysis with an F40S hi flux polysulfone dialyzer to rapidly clear vancomycin from a pediatric patient in acute oliguric renal insufficiency. Previous reports of vancomycin intoxication managed with high-efficiency dialysis membranes have reported vancomycin removal rates between 60 and 79 percent [11, 14]. Removal of vancomycin in our pediatric case was approximately 75 percent.

## 4. Conclusion

The current recommendations to target higher serum levels of vancomycin are likely to result in an increased incidence of adverse effects, including unintended drug accumulation and nephrotoxicity. Our report emphasizes the importance of vigilant monitoring of vancomycin levels and renal function with high-dose vancomycin and demonstrates the safety, efficacy, and superiority of high-flux hemodialysis as a method to remove extremely elevated vancomycin concentrations from the intravascular space. We were able to remove 75.68% of vancomycin within a 6-hour period (213.2 mcg/mL to 51.8 mcg/mL) without any adverse effects, with full recovery of renal function, and preservation of hearing. High-flux hemodialysis should be considered as a viable therapy in children with vancomycin toxicity and concurrent renal insufficiency.
